# Solitary Extramedullary Plasmacytoma of the Cricoid Cartilage—Case Report

**DOI:** 10.3389/fonc.2017.00284

**Published:** 2017-11-27

**Authors:** Simone Krebs, Ian Ganly, Ronald Ghossein, Joanna Yang, Joachim Yahalom, Heiko Schöder

**Affiliations:** ^1^Department of Radiology, Molecular Imaging and Therapy Service, Memorial Sloan Kettering Cancer Center, New York, United States; ^2^Department of Surgery, Head and Neck Service, Memorial Sloan Kettering Cancer Center, New York, United States; ^3^Department of Pathology, Memorial Sloan Kettering Cancer Center, New York, United States; ^4^Department of Radiation Oncology, Memorial Sloan Kettering Cancer Center, New York, United States

**Keywords:** plasmacytoma, larynx, cricoid, radiotherapy, ^18^F-FDG-PET/CT

## Abstract

Solitary plasmacytoma (SP) is an extremely rare form of hematologic malignancy that can be classified as solitary bone plasmacytoma or solitary extramedullary plasmacytoma. Here, we report a patient who presented with progressive shortness of breath and foreign body sensation in his throat. Fluorodeoxyglucose positron emission tomography/computed tomography (^18^F-FDG-PET/CT) demonstrated an abnormal FDG-avid soft tissue mass arising from the larynx involving the cricoid cartilage without additional suspicious lesions. Histology revealed an abundance of plasma cells; immunohistochemistry was positive for CD138 expression and lambda chains, and negative for CD20. Comprehensive imaging studies and panendoscopy of the ENT tract confirmed solitary disease involvement. Following additional systemic work-up, a diagnosis of extramedullary plasmacytoma was rendered. The patient underwent definitive radiotherapy using intensity-modulated radiation therapy (total dose of 46 Gy, divided in 23 fractions of 200 cGy). Serial PET/CT showed the stepwise resolution of abnormal FDG uptake and resolution of the cricoid cartilage lesion. With 22 months of follow-up, the patient remains free of disease. We describe the rare case of SP presenting as a FDG-avid hypermetabolic soft tissue mass in the cricoid cartilage, which should be considered in the differential diagnosis of laryngeal tumors. Of note, SP is radiosensitive; favorable outcome can be expected once treated with doses of 40–50 Gy. FDG PET/CT is helpful in diagnosis and response assessment for this disease.

## Introduction

Multiple myeloma (MM), a malignant plasma cell tumor, accounts for approximately 1.8% of all cancers and 15% of hematologic malignancies (1–3). Solitary plasmacytoma (SP), an extremely rare form within this entity that accounts for approximately 4% of plasma cell malignancies (4), can be classified as solitary bone plasmacytoma (SBP) or solitary extramedullary plasmacytoma (SEMP). Here, we report the rare case of a laryngeal SEMP involving the cricoid cartilage.

## Background

**Figure 1:**
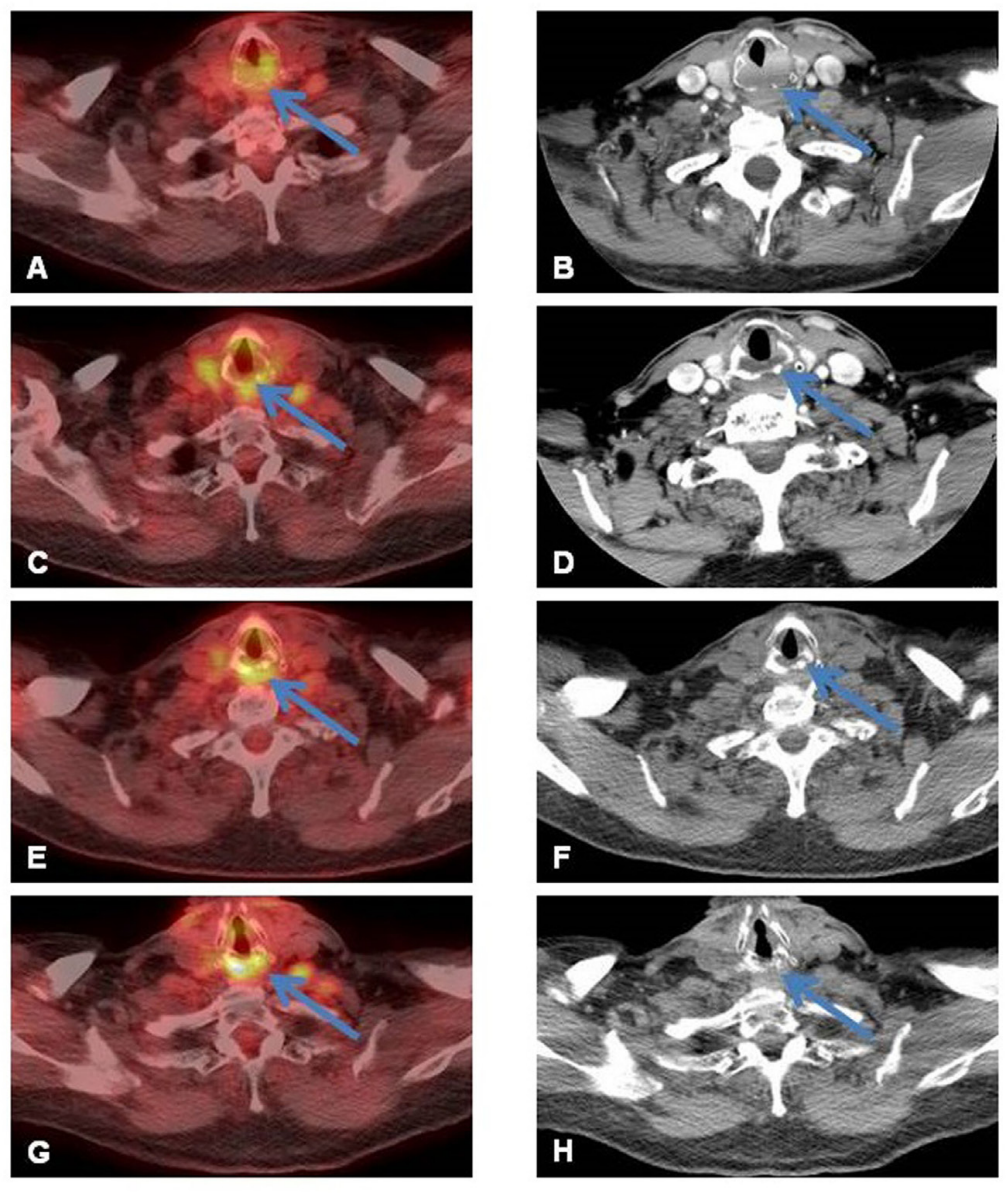
**(A)** Focal increased FDG uptake [maximum standard uptake value (SUV) of 3.8] in the larynx noted on axial fused PET/CT image (blue arrow) corresponds to a **(B)** soft tissue mass (3.3 cm × 1.7 cm × 2.2 cm) in the posterior cricoid cartilage on contrast-enhanced (CE)-CT scan of the larynx (blue arrow). **(C)** Posttreatment follow-up FDG-PET/CT acquired 2 months after the end of the radiation therapy showed a decrease of the intensity of uptake; persistence of a very low diffuse uptake (SUV 4.9) is noted probably reflecting post-radiation changes (blue arrow). **(D)** CE-CT of the neck confirmed decreased size of the residual lesion, now measuring 2.5 cm × 1.0 cm × 2.0 cm (transverse by AP by craniocaudal) (blue arrow) and improved narrowing of the infraglottic airway. **(E)** Follow-up PET performed 6 months later showed persistent non-specific uptake (SUV 4.7) and **(F)** essentially unchanged size of the residual soft tissue on non-contrast, low-dose CT of the PET/CT. **(G)** One-year follow-up PET/CT demonstrated non-specific uptake, and **(H)** on corresponding non-contrast, low-dose CT of the PET/CT, normal appearance of the cricoid without residual soft tissue was seen.

**Figure 2:**
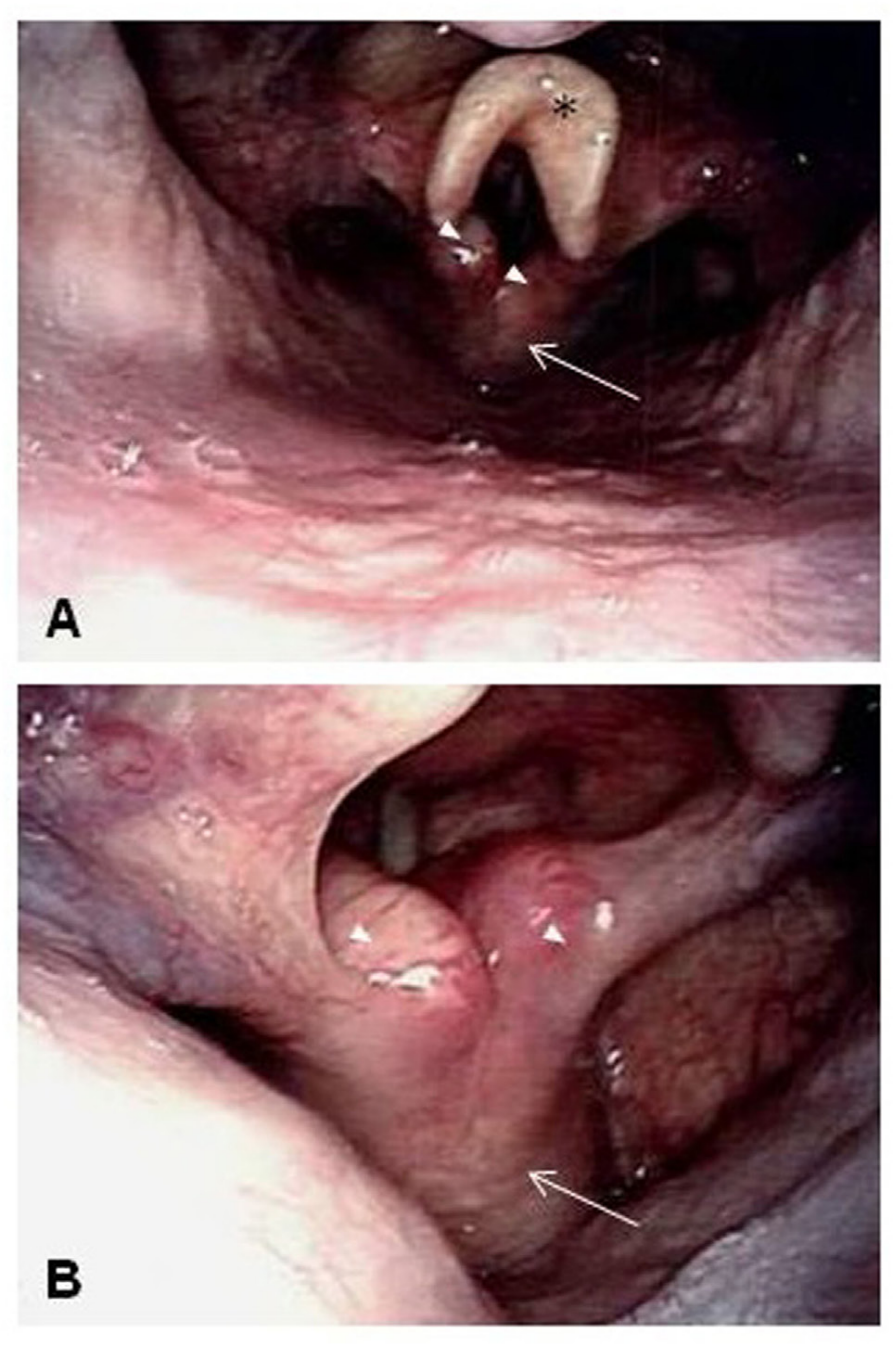
Laryngoscopy confirmed the diffuse enlargement of the cricoid cartilage with circumferential subglottic narrowing. **(A)** The epiglottis (black asterisk), arytenoid cartilages (white arrow heads), and enlarged cricoid cartilage (white arrow). **(B)** A magnified view of the enlarged cricoid cartilage (white arrow) and arytenoid cartilages (white arrow heads).

**Figure 3:**
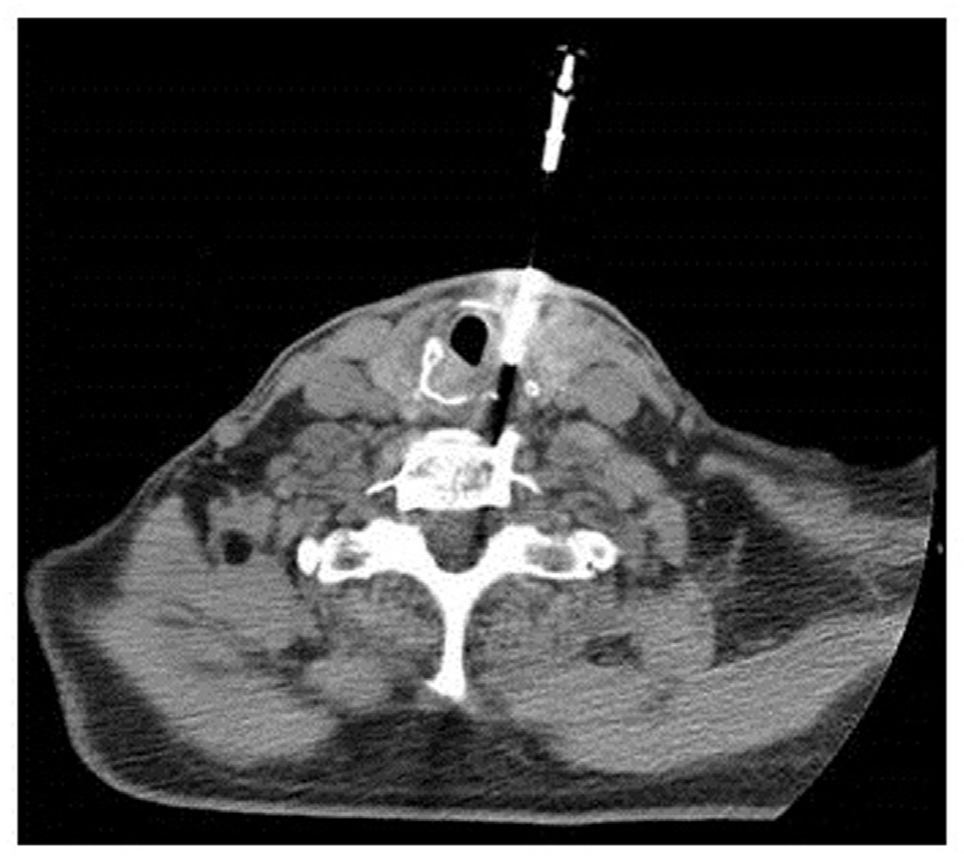
CT-guided biopsy of the mass in the cricoid cartilage.

A 77-year-old man who grew up in Sicily presented with progressive shortness of breath and foreign body sensation in his throat to an outside emergency department while traveling to Arizona. Other symptoms included intermittent cough of whitish sputum, intermittent wheezing, and dyspnea on exertion. Outside chest X-ray and CT of the chest revealed thickened soft tissue beneath the vocal cords. A prior chest CT from April 2014 showed no laryngeal lesion. A dedicated CT of the larynx performed in March 2015 with intravenous contrast then demonstrated an expansile soft tissue mass arising in the posterior half of the cricoid cartilage with mild airway narrowing measuring 3.3 cm × 1.7 cm × 2.2 cm. No infiltration of adjacent soft tissues, trachea, or arytenoid cartilage was detected, nor was focal calcification observed (Figure 1B, blue arrow). FDG-PET performed 4 days later showed the soft tissue mass involving the posterior half of the cricoid cartilage with increased FDG uptake [maximum standard uptake value (SUV): 3.8] (Figure 1A, blue arrow). No other sites of abnormal increased FDG uptake were identified elsewhere in the body. Possible differential diagnosis included primary lesions of the cartilage (chondroma versus chondrosarcoma), lymphoma/lymphoid hyperplasia, and an inflammatory process. The next day, laryngoscopy confirmed the diffuse enlargement of the cricoid cartilage with circumferential subglottic narrowing (Figure 2). Figure 2A shows the epiglottis, arytenoid cartilage, and enlarged cricoid cartilage. Figure 2B shows a magnified view of the enlarged cricoid cartilage. Since an attempted endoscopic biopsy was not diagnostic, a CT-guided biopsy was obtained four weeks later (Figure 3).

**Figure 4:**
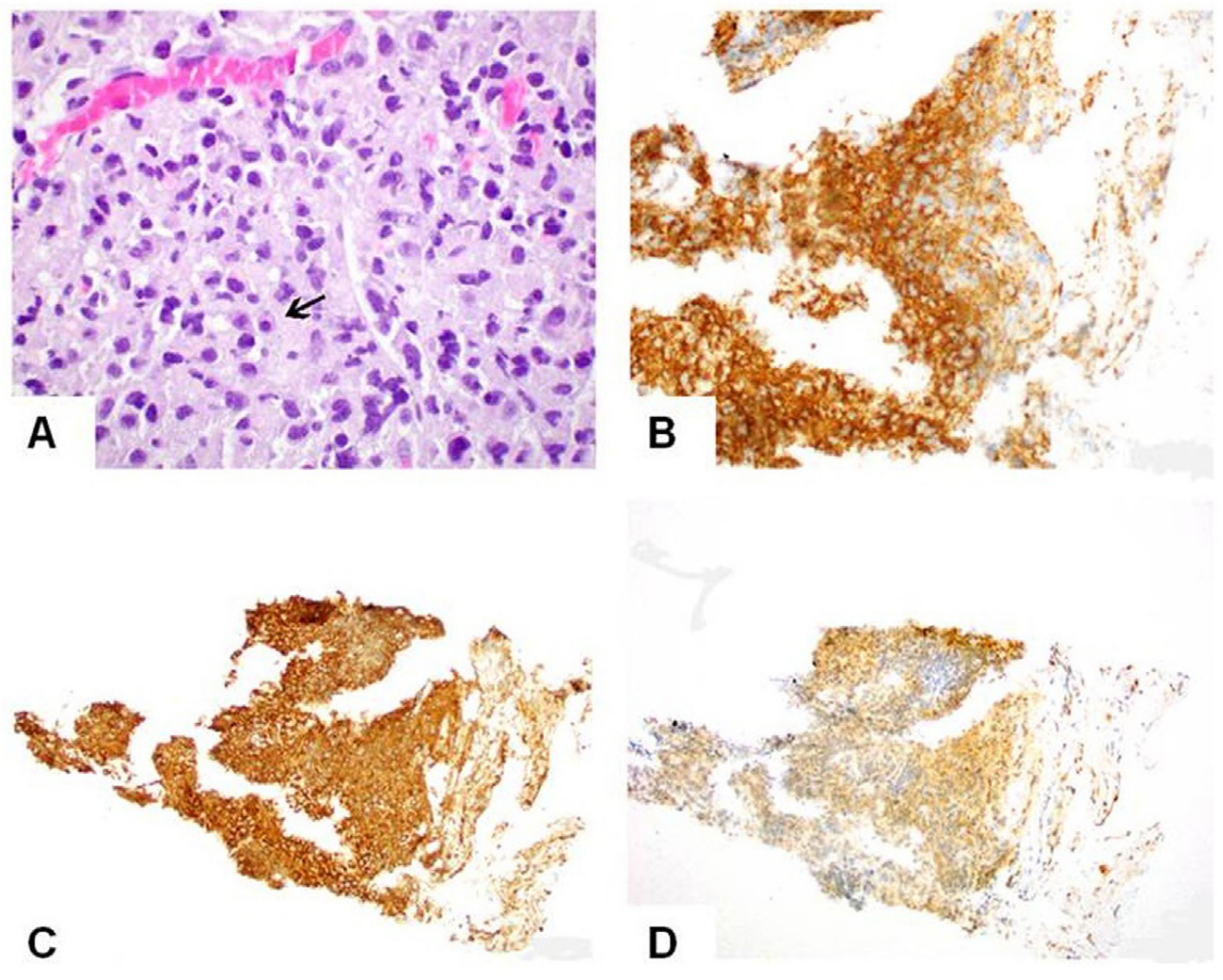
Microscopic pictures of the plasmacytoma. **(A)** The tumor is entirely composed of plasma cells, some showing the typical perinuclear clearing associated with Golgi apparatus (arrow), H&E, 600×. **(B)** Immunostain for CD138 shows positive (golden brown) staining in the tumor (200×). **(C)** Immunostains for lambda and **(D)** kappa light chains show a significant difference in staining intensity between kappa and lambda chains in favor of lambda chains (200×) demonstrating that the tumor is lambda-restricted.

Histologically, the tumor was entirely composed of plasma cells, some of which showed the typical perinuclear clearing associated with the Golgi apparatus (Figure 4A, arrow). On immunohistochemistry, the neoplastic cells stained positive for CD138 (Figure 4B) and stains for immunoglobulin lambda (Figure 4C) and kappa (Figure 4D) in favor of lambda chains, demonstrating that the tumor is lambda light chain restricted. Complete lack of CD20 expression excluded an aggressive B-cell lymphoma. A diagnosis of extramedullary plasmacytoma (EMP) was rendered. Additional systemic work-up including bone marrow biopsy, urine/serum protein electrophoresis, immunofixation, and blood chemistry showed no evidence of MM, leading to the final diagnosis of SEMP.

The patient underwent definitive radiotherapy using intensity-modulated radiation therapy (total dose of 46 Gy, divided in 23 fractions of 200 cGy; completed in July 2015). He tolerated the treatment well; the acute toxicity noted was grade 1 voice change. He had no unanticipated events during his RT course; however, he developed likely radiation-induced subclinical hypothyroidism (TSH 9.5, freeT4 normal), for which he was treated with levothyroxine. This RT-related complication is not unexpected in view of the thyroid’s immediate vicinity to the larynx; therefore, baseline thyroid function tests prior to RT initiation and subsequent continued testing should be considered to detect transient or permanent dysfunction (5). Serial follow-up imaging studies showed stepwise resolution (Figure 1C–H, arrows). With 26 months of follow-up, the patient remains free of disease.

## Discussion

Solitary extramedullary plasmacytoma is defined as a solitary soft tissue lesion with clonal plasma cells, an absence of bone marrow involvement, and an absence of end-organ damage secondary to lympho-plasma cell proliferative disorder (6). SEMP occurs most commonly in the head and neck (4, 6), demonstrating a predilection for male gender and for pharynx (21.5%), nasal cavity (19.3%), oral cavity (14.7%), and paranasal sinuses (13.0%) (6). Involvement of the larynx, however, is rare, with a total of 32 cases (4.7%) reported in the SEER database from 1973 to 2000 (6). The clinical presentation in patients with laryngeal involvement is varying and dependent on location and lesion size, usually involving hoarseness and/or dysphagia, sometimes even acute airway compromise requiring emergent bronchoscopic resection have been reported (7). In our literature search, we found only four case reports that have described SEMP involving the cricoid cartilage (7–10). Due to the low incidence of SP, reliable data regarding the clinical course and outcome of different treatment approaches are lacking. SP is radiosensitive (11, 12), with favorable outcomes when treated with doses of 40–50 Gy (11). Other treatment options include surgical resection, laser excision, or combined modality therapy (12, 13). SEMP, in particular for sites in the head and neck, carries a better prognosis than SBP, regardless of tumor grade or stage (6). Disease progression to MM is less common in SEMP as compared to SBP (36 vs. 53%) (14). Risk factors for progression include age >60 (12) and angiogenesis (15). Progression to MM and amyloid deposition has been associated with poor prognosis (13).

Contemporary imaging studies for exclusion of MM and thus confirmation of SP, include whole-body low-dose computed tomography (to identify lytic osseous lesions), MRI of spine and pelvis, and FDG PET/CT (PET/CT) as second-line imaging techniques (16). PET is inferior to MRI for detection of bone marrow involvement in advanced (stage III) MM (17) or recurrent MM (18), but it is a useful tool for evaluating treatment response and for prognostication (18–20). In the current case, serial PET/CT showed the stepwise resolution of abnormal FDG uptake and resolution of the cricoid cartilage lesion following intensity-modulated radiation therapy. In contrast to MM, data on the role of FDG PET-CT in SP are scarce (see Table S1 in Supplementary Material for a summary of reported cases of plasmacytoma in head and neck imaged by PET/CT). In one study (21) of 23 patients with SP (including SBP and SEMP) with 54 tumor sites, only 4 lesions were confined to soft tissue areas (head and neck, breast, and pelvis), and 50 were bone lesions. Per number of analyzed anatomical sites, the sensitivities, and the specificities of FDG PET/CT vs MRI were 98 and 99% vs 93 and 94%, respectively. However, in 50% of patients, the FDG PET/CT detected plasmacytoma lesions in 18 areas that were outside the field of view for the MRI. In follow-up, MRI showed six false positive findings, which were correctly classified as scar tissue on PET.

The histologic differential diagnoses of a monoclonal cell population with plasmacytic features includes plasma cell neoplasms, such as plasmacytoma, plasma cell myeloma, monoclonal gammopathy of uncertain significance, and smoldering myeloma (22), as well as other B cell lymphomas such as extranodal marginal zone lymphoma and lymphoplasmacytic lymphoma (23). Analysis of adhesion molecule and chemokine receptor CD49d and CXCR4 may be helpful in differentiating SP from MM (24). Nevertheless, comprehensive imaging studies and panendoscopy of the ear, nose, and throat tract are necessary to confirm solitary disease involvement (4, 13, 21).

The etiology of SEMP remains unknown; however, viral pathogens (particularly Epstein–Barr virus) and chronic irritation from inhaled irritants associated with its diagnosis have been reported (25). While specific chromosomal anomalies have been identified in MM (26), this has not been demonstrated in SBP or SEMP.

## Concluding Remarks

We describe the rare case of SP presenting as an FDG-avid hypermetabolic soft tissue mass in the cricoid cartilage, which should be considered in the differential diagnosis of laryngeal tumors. Whereas updated guidelines for treatment, risk stratification, and prognostic criteria for patients with MM have been established, determination of a concise approach for patients with SP has been hampered, largely due to the rare occurrence and limited literature. Of note, SP is radiosensitive; favorable outcome can be expected once treated with doses of 40–50 Gy. FDG PET/CT is helpful in diagnosis and response assessment for this disease.

## Ethics Statement

Written informed consent was obtained from the patient for publication of this case report and accompanying images.

## Author Contributions

SK and HS assembled, analyzed, and interpreted the patient’s imaging data. IG performed the biopsy. RG undertook the histologic analyses. JaY and JmY provided the treatment and continue with the patient’s follow-up. All authors contributed to writing the manuscript. All authors read and approved the final manuscript.

## Conflict of Interest Statement

The authors declare that the research was conducted in the absence of any commercial or financial relationships that could be construed as a potential conflict of interest.

## Abbreviations

SP, solitary plasmacytoma; SBP, solitary bone plasmacytoma; SEMP, solitary extramedullary plasmacytoma; 18F-FDG-PET/CT, fluorodeoxyglucose positron emission tomography/computed tomography; EMP, extramedullary plasmacytoma; SUV, standard uptake value; MM, multiple myeloma.
