# Supplementary material for: Solitary Extramedullary Plasmacytoma of the Cricoid Cartilage—Case Report
Source: Front Oncol. 2017 Nov 27;7:284. doi: 10.3389/fonc.2017.00284 (PMC5711767; doi:10.3389/fonc.2017.00284)
Supplement: Supplementary file 1 [file Data_Sheet_1.docx]

Supplementary Material

**Solitary Extramedullary Plasmacytoma of the Cricoid Cartilage –**

**Case Report**

Simone Krebs^*^, Ian Ganly, Ronald Ghossein, Joanna Yang, Joachim Yahalom, Heiko Schöder

*** Correspondence:** Corresponding Author: krebss@mskcc.org

# Supplementary Table

**Supplementary table 1.** Summary of reported cases of plasmacytoma in head and neck imaged by PET/CT.

**References**

1. R. Fernandez Lopez, I. Borrego Dorado, A. Paz Coll, R. Vazquez Albertino, P. Gomez Camarero and S. Sanz Viedma: [18F-FDG PET-CT in a case of solitary plasmacytoma of the soft palate]. *Rev Esp Med Nucl*, 29(5), 263-5 (2010) doi:10.1016/j.remn.2010.02.010

2. M. J. Dong, Z. F. Liu, G. L. Wang, K. Zhao, M. L. Sun, H. T. Wang and S. Y. Yang: A low F-18 FDG uptake in the nasopharynx plasmacytoma detected by FDG PET/CT. *Clin Nucl Med*, 36(11), 1053-5 (2011) doi:10.1097/RLU.0b013e31821a2ae7

3. A. Yoshida, S. Borkar, B. Singh, R. A. Ghossein and H. Schoder: Incidental detection of concurrent extramedullary plasmacytoma and amyloidoma of the nasopharynx on [18F]fluorodeoxyglucose positron emission tomography/computed tomography. *J Clin Oncol*, 26(35), 5817-9 (2008) doi:10.1200/jco.2008.18.6338

4. T. Kato, E. Tsukamoto, T. Nishioka, A. Yamazaki, H. Shirato, S. Kobayashi, M. Asaka, M. Imamura and N. Tamaki: Early detection of bone marrow involvement in extramedullary plasmacytoma by whole-body F-18 FDG positron emission tomography. *Clin Nucl Med*, 25(11), 870-3 (2000)

5. G. Treglia, G. Paone, A. Meyer, L. Ceriani and L. Giovanella: An Unusual Case of Extramedullary Plasmacytoma of the Hypopharynx Detected by (18)F-FDG PET/CT. *Nucl Med Mol Imaging*, 48(4), 328-9 (2014) doi:10.1007/s13139-014-0271-7

6. V. Ravo, M. G. Calvanese, R. Manzo, M. G. Cuomo, F. Cammarota, P. Murino and P. Muto: Solitary plasmacytoma of the larynx treated with radiotherapy: a case report. *Tumori*, 98(2), 35e-38e (2012) doi:10.1700/1088.11945

7. Y. Z. Alabed, R. Rakheja and J. Laufer: Solitary extramedullary plasmacytoma of the parotid gland imaged with 18F-FDG PET/CT. *Clin Nucl Med*, 39(6), 549-50 (2014) doi:10.1097/RLU.0b013e3182a75c95

8. S. Shahani, A. Ahmad, F. H. Barakat, H. H. Chuang, N. H. Fowler, J. N. Myers, C. Stava and M. A. Habra: F-18 FDG PET/CT detecting thyroid plasmacytoma after the successful treatment of gastric large B-cell lymphoma. *Clin Nucl Med*, 36(4), 317-9 (2011) doi:10.1097/RLU.0b013e31820aa079
