# Supplementary material for: Solitary Extramedullary Plasmacytoma of the Cricoid Cartilage—Case Report
Source: Front Oncol. 2017 Nov 27;7:284. doi: 10.3389/fonc.2017.00284 (PMC5711767; doi:10.3389/fonc.2017.00284)
Supplement: Supplementary file 2 [file Data_Sheet_2.pdf]

Supplementary table 1. Summary of reported cases of plasmacytoma in head and neck imaged by PET/CT. Abbreviations: **F**: female; **M**: male; **RT**: radiotherapy; **EBRT**: external beam radiation therapy; **N/A**: not available

Reference	Pat. ID	Sex	Age (yrs)	Site	Size (mm)	SUVmax (PET baseline)	MRI / CT neck	Treatment	Follow-up imaging (Y/N)	Resolved (Y/N)
				**Oral cavity**						
Fernandez-Lopez (2010)[1]	1	F	61	Soft palate	17×14×20	6.7	MRI, CT	RT	N/A	N/A
				**Nasopharynx**						
Dong (2011)[2]	1	N/A	N/A	Nasopharynx	71x33	1.4	N/A	N/A	N/A	N/A
Yoshida (2008)[3]	1	F	52	Nasopharynx	16x12	14.9	N/A	Surgery	N/A	N/A
Kato (2000)[4]	1	F	34	Nasopharynx	N/A	19.5	MRI	RT	Y	Y
				**Hypopharynx**						
Treglia (2014)[5]	1	F	69	Hypopharynx	N/A	3.1	CT	Surgery	N/A	N/A
				**Larynx**						
Present case	1	M	77	Cricoid cartilage	33x17x22	3.8	MRI, CT	RT (46 Gy)	Y	Y
Ravo (2012)[6]	1	M	56	False vocal cord and arytenoid cartilage	N/A	4.5	CT	CO_2 laser +RT (50Gy)	Y	Y
				**Other sites**						
Alabed (2014)[7]	1	F	75	Parotid gland	39x29	30	CT	RT	N/A	N/A
Shahani (2011)[8]	1	M	82	Thyroid gland	45x30	18.8	N/A	Surgery, EBRT + rituximab	Y	Y
